# *Dirofilaria immitis* is endemic in rural areas of the Brazilian Amazonas state capital, Manaus

**DOI:** 10.1590/S1984-29612023018

**Published:** 2023-04-28

**Authors:** Ulysses Carvalho Barbosa, Alessandra Ferreira Dales Nava, José Vicente Ferreira, Cindy Alves Dias, Viviane Costa da Silva, Hugo Guimarães de Mesquita, Raquel Telles de Moreira Sampaio, Wanilze Gonçalves Barros, Emanuelle de Sousa Farias, Tullio Romão Ribeiro da Silva, James Lee Crainey, Wanderli Pedro Tadei, Hector Henrique Ferreira Koolen, Felipe Arley Costa Pessoa

**Affiliations:** 1 Programa de Pós-graduação em Biodiversidade e Biotecnologia PPG-Bionorte, Manaus, AM, Brasil; 2 Laboratório de Etnoepidemiologia, Coordenação de Sociedade Ambiente e Saúde, Instituto Nacional de Pesquisas da Amazônia, Manaus, AM, Brasil; 3 Laboratório de Ecologia Transmissíveis na Amazônia, Instituto Leônidas e Maria Deane, Manaus, AM, Brasil; 4 Programa de Pós-graduação em Entomologia, Instituto Nacional de Pesquisas da Amazônia, Manaus, AM, Brasil; 5 Programa de Pós-graduação em Biointeração Parasita-Hospedeiro, Instituto Leônidas e Maria Deane, Manaus, AM, Brasil; 6 Laboratório de Malária e Dengue, Coordenação de Sociedade Ambiente e Saúde, Instituto Nacional de Pesquisas da Amazônia, Manaus, AM, Brasil; 7 Laboratório de Química Bioorgânica do Grupo de Metabolômica e Espectrometria de Massas da UEA, Manaus, AM, Brasil

**Keywords:** Dirofilaria immitis, Dirofilariasis, Urban expansion, zoonoses, Manaus, Amazonia, Dirofilaria immitis, Dirofilariose, Expansão urbana, zoonoses, Manaus, Amazônia

## Abstract

The canine filarial parasite *Dirofilaria immitis* has not been reported in Brazil´s Amazonas state capital, Manaus, for over a century. Here, we report one imported and 27 autochthonous *D. immitis* infections from a microfilarial survey of 766 domestic dog blood samples collected between 2017 and 2021 in Manaus. An Overall prevalence estimate of 15.44% (23/149) was calculated from our two rural collection sites; a prevalence of 1.22% (4/328) was estimated at our periurban collection site, and an overall prevalence of 0.35% (1/289) was calculated from our two urban clinic collections. Our data suggest that in the urban areas of Manaus, where the parasites are very likely vectored by the same species of mosquito that historically vectored *Wuchereria bancrofti* (*Culex quinquefasciatus*), prevalence levels are very low and possibly maintained by an influx from rural areas where sylvatic reservoirs and/or more favorable vector transmission dynamics maintain high prevalences.

## Resumo

O parasita filarial canino, *Dirofilaria immitis,* causa doença zoonótica, mas não tem sido registrado em Manaus, capital do estado do Amazonas, há mais de um século. Neste trabalho, foi relatado uma infecção por *D. immitis* alóctone e 27 autóctones de um levantamento de microfilárias em 766 amostras de sangue em cães domésticos, coletados entre 2017 e 2021 em Manaus. A prevalência de 15,44% (23/149) foi estabelecida em áreas rurais, 1,22% (4/328) para áreas periurbanas e de 0,35% (1/289) para duas clínicas veterinárias localizadas na zona urbana da cidade. Estes dados sugerem, portanto, que nas áreas urbanas de Manaus, nas quais o parasita é provavelmente vetorizado pela mesma espécie de mosquito que, historicamente, transmitiu *Wuchereria bancrofti* (*Culex quinquefasciatus*). Também os níveis de prevalência são baixos e, possivelmente, mantidos por um influxo de áreas rurais onde reservatórios silvestres e/ou dinâmicas de transmissão vetorial mais favoráveis mantêm uma prevalência mais elevada.

Dirofilariosis is a zoonotic filarial disease caused by two species of parasite from the genus *Dirofilaria: Dirofilaria immitis* and *Dirofilaria repens* (Dantas-Torres et al., 2017). The two parasite species cause differing pathologies and have differing epidemiologies and global distributions. In Latin America, the disease is caused almost exclusively by *Dirofilaria immitis*, which principally infects domestic dogs but can also cause patent spill-over infections in domestic cats and other wild animals (Dantas-Torres et al., 2017; Dantas-Torres & Otranto, 2020).

Companion animal owners in endemic areas of North America often use macrocyclic lactones (typically ivermectin) as prophylactics to prevent their animals contracting *D. immitis* infections and can stay abreast of the latest treatment options through information provided by local heart worm societies (Simón et al., 2012). Although *D. immitis* infections cannot cause patent infections in humans, partially developed (dead) larvae can appear on X-rays and CT scans of human lungs as coin-like lesions (Simón et al., 2012; McCall et al., 2008). An accurate knowledge of the distribution of these parasites can thus potentially help shorten clinical investigations and in this way reduce health service clinical spending in endemic areas. Despite the potential veterinary and public health benefits that can come from accurate and up-to-date *D. immitis* disease mapping, however, parasite surveys are rarely performed in Latin America. Thus, although the presence of *D. immitis* has been confirmed in a number of municipalities surrounding the state capital of Manaus (Soares et al., 2014; Silva et al., 2008), there have been no accounts of *D. immitis* in Manaus for more than 100 years (Gordon & Young, 1922). Given the enormous geographical and populational expansion of Manaus over the last century and indeed the apparent loss of two formally endemic filarial parasites (*Mansonella ozzardi* and *Wuchecria bancrofti*), there was thus a clear and urgent need for an update on the distribution of these parasites in the city (Abrahim et al., 2019; Costa et al., 2023; Martins et al., 2021).

**Figure 1:**
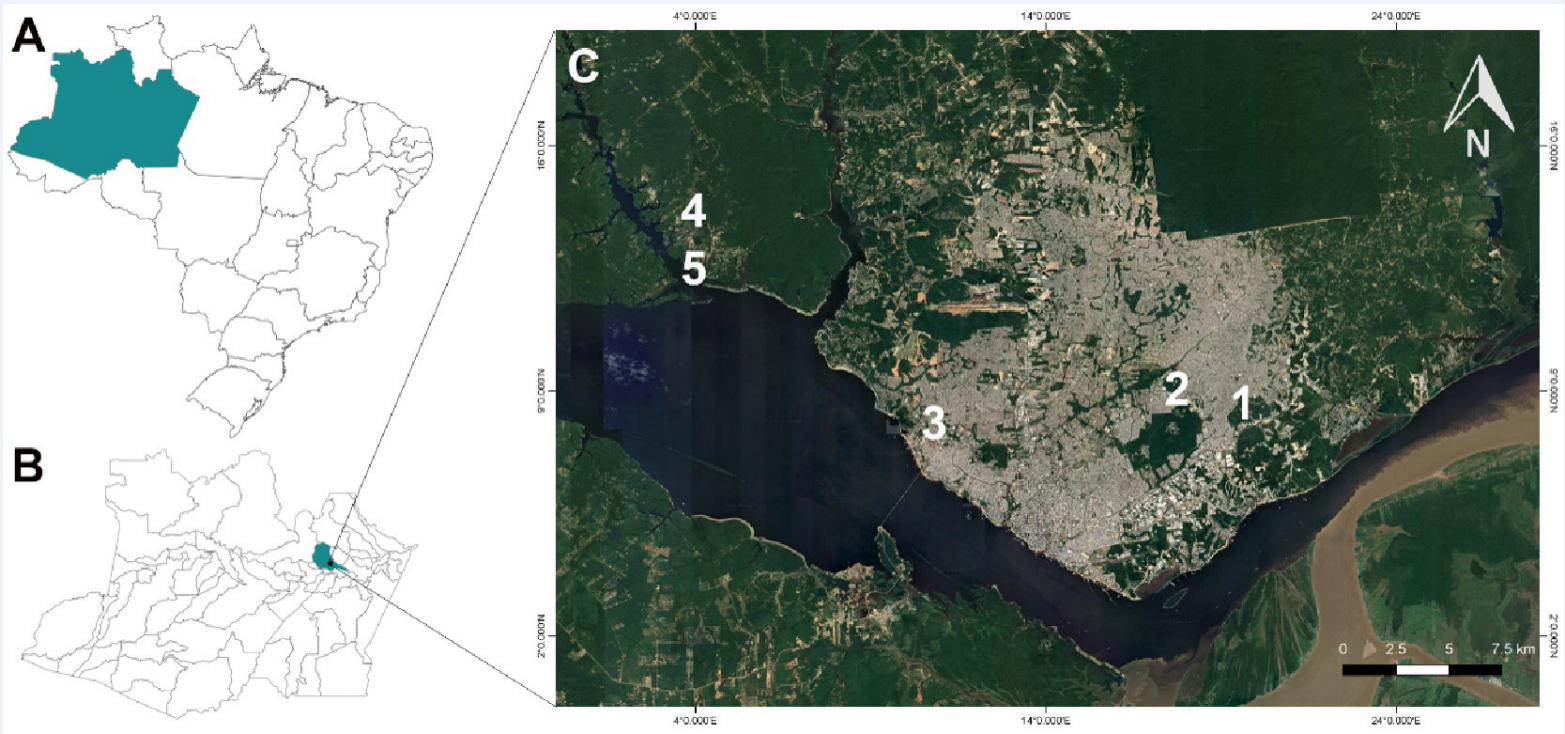
Distribution of sampled locations in the urban and rural regions of the city of Manaus. **A**: A map of Brazil; **B**: A map of Amazonas state; **C**: The distribution of five sample locations within and around Manaus: **1.** Mobile Castration Unit (MCU) **2.** Veterinary Clinic 1 (VC1); **3**. Veterinary Clinic 2 (VC2); **4.** Nossa Senhora do Livramento Community; **5.** Our Lady of Fatima Community.

To investigate the current status of Dirofilariosis in and around the city of Manaus, canine blood surveys were carried out at: urban veterinary clinics within the city; a veterinary clinic on the city´s outskirts and at two rural settlements just outside the city. The urban surveys were performed in partnership with two city veterinary clinics: a Mobile Castration Unit of the Municipal Health Department headquartered in the heart of the city (03°04'45”S 59°55'59”W) and known in Brazil as SEMSA and a private veterinary clinic named here as VC1 (See Figure 1). As can be seen in Figure 1, VC1 is located in the east of the city (03 °04'27”S 59°57'39”W), in a densely populated, low-income neighborhood which we have classified as urban. A second private veterinary clinic (named here as VC2) located in the west of the city (03°05'07”S 60°03'49”W) was also included. As can be seen in Figure 1, VC2 is located on the periphery of Manaus in a region that contains many family farms and which is much less densely populated than the area which VC1 is situated. For this reason, we have classified VC2 as being in a periurban area. Our rural blood surveys were done in partnership with the “Nossa Senhora do Livramento Community” (02°59'57”S 60°11'57”W) and the “Nossa Senhora de Fátima Community” (03°01'10”S 60°09'55”W). Both these communities are located in the Tupé Sustainable Development Reserve, on the north bank of the Rio Negro and contain large areas of ​​native vegetation and an unpolluted water source supplied by the Tarumã-Mirim River. Both these areas also have abandoned lands that allow for the accumulation of stagnant water and mosquito breeding.

**Figure 2:**
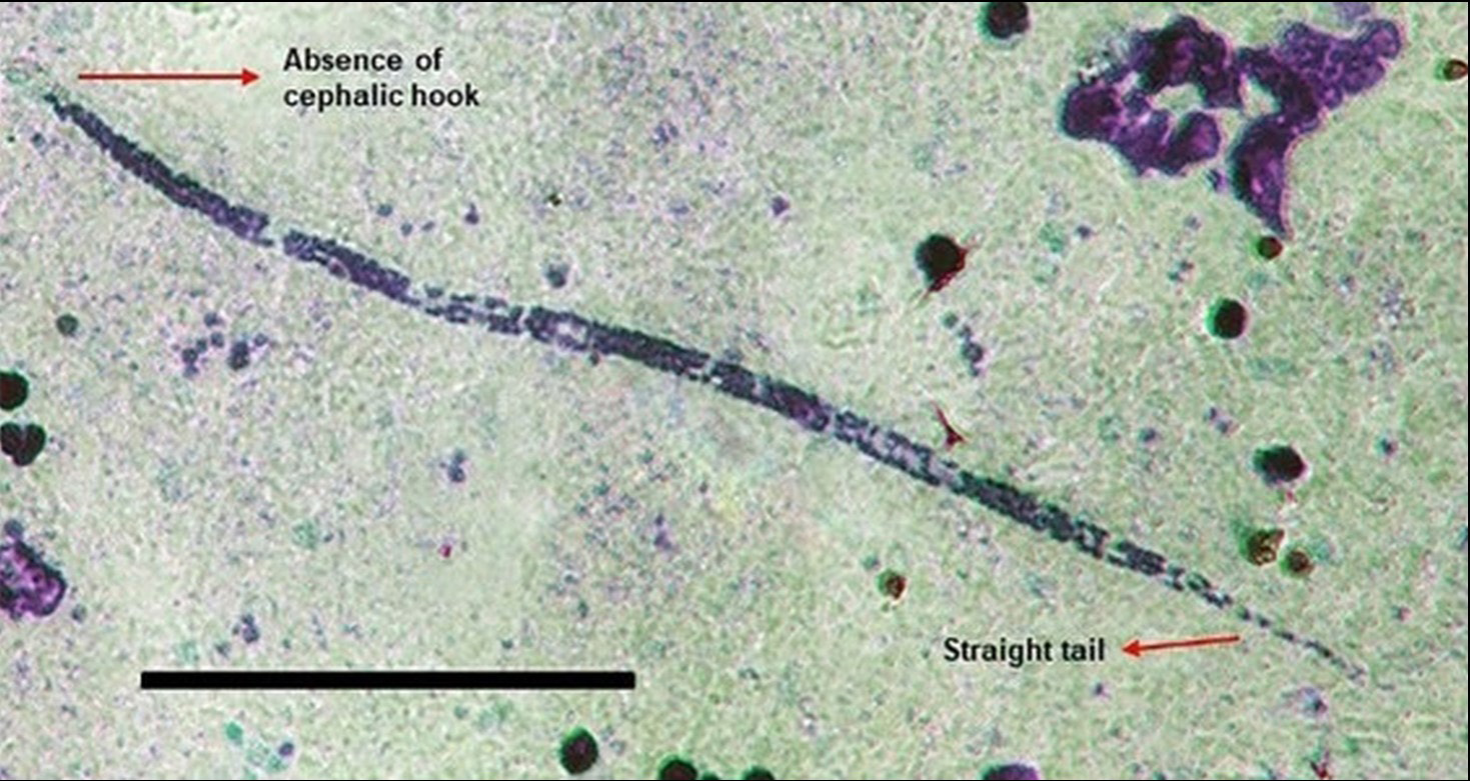
Canine filarial parasite *Dirofilaria immitis* from Manaus. Shows a *D. immitis* microfilariae image captured with a JVC KY-F55B digital camera attached to a light microscope using a 40X objective. The scale bar is 100 μm in length; identifying morphological features are indicated.

Convenience sampling was conducted at all of the sampling areas. Urban collections made from the Mobile Castration Unit were performed between November 2016 and February 2017 and at the VC1 clinic between December 2017 and March 2018. The periurban collections at VC2 were made from May 2018 to December 2018. Rural collections were made between May 2019 and November 2019 in the Nossa Senhora do Livramento Community and in December 2021 at the Nossa Senhora de Fátima Community. Blood samples were drawn from adult dogs (aged between 2 and 15 years) by cephalic vein puncture, using a sterile needle and vacutainer tube containing EDTA. The collection tubes were labelled with unique identifiers, then placed in an isothermal box and taken to the Ethnoepidemiology Laboratory of the National Institute for Research in the Amazon (INPA) or the Leonidas and Maria Deane Institute (Fiocruz Amazônia) for analysis. At same time blood samples were taken, basic information about the dogs´ lives was collected from the animal´s owners, with specific attention being paid to where the animal had been born and resided. To visualize the microfilariae, whole blood samples were used to make thick blood smears. These were prepared using the method described by Knight (1977) in which two drops of blood (approximately 40 µl) are applied to the slide and allowed to dry at room temperature for 24 hours before they are stained with methylene blue and Giemsa and visualized with a light microscope using 10x and 40x objectives. Microfilariae were identified morphologically following Peroba et al. (2022); an example of one of the parasites that was encountered is shown in Figure 2.

In total we examined the blood of 766 animals: 287 males and 479 females. The sampling was performed as follows: 238 dogs from the Mobile Castration Unit; 51 dogs from VC1 site; 328 dogs from VC2 site; 73 dogs from the Nossa Senhora do Livramento Community and 76 dogs from the Nossa Senhora de Fátima Community. Of all the animal surveyed just 28 were positive for *D. immitis* of which only one was not born and raised in Manaus (having arrived in the city from Belem) giving our survey an overall *D. immitis* prevalence rate of 3.66% (28/766). Table 1 provides a summary of the *D. immitis* blood survey prevalence levels calculated for each of the five individual collections performed for this study. From this data it can be observed that our rural collections had the highest *D. immitis* prevalence levels of 15.44% (23/149) whereas our urban collections had the lowest prevalence levels corresponding to 0.35% (1/289) and that our periurban site had an intermediary prevalence level of 1.22% (4/328). It can also be seen in Table 1 that, consistent with previous studies and expectations (Dantas-Torres et al., 2017; McCall et al., 2008; Simón et al., 2012), our study detected more *D. immitis* infections in male dogs 16/271 (5.51%) than it detected in female dogs 12/467 (2.56%).

**Table 1:** Prevalence of *Dirofilaria immitis* among domestic dogs, as determined by light microcopy examination of canine blood smears taken at five surveying localities within and around Manaus (Amazonas, Brazil).

Locality	Sampled dogs		Positive dogs		Total
	Males	Females	Males	Females	
MCU	74	164	1 (1.35%)	0 (0%)	238 (0.4%)
VC1	25	26	0 (0%)	0 (0%)	51 (0%)
VC2	114	214	2 (1.75%)	2 (0.9%)	328 (1.2%)
Livramento	34	39	9 (26.5%)	2 (5.1%)	73 (15.1%)
Fátima	40	36	4 (10%)	8 (22.2%)	76 (15.8%)
Total	287	479	16 (5.6%)	12 (2.5%)	766 (3.7%)

MCU: mobile castration unit; VC1: veterinary clinic 1; VC2: veterinary clinic 2.

In the Gordon & Young (1922) study, they sacrificed 50 stray dogs and nine stray cats for a veterinary parasite survey in Manaus. Their necropsies found adult *D. immitis* in the hearts of two of the dogs they killed, as well as a second unidentified species of filarial parasite in the peritoneal of a third, and thus showed that ≥4% of the stray dogs that they surveyed were infected with *D. immitis*. Unfortunately, the blood survey carried out by Gordon & Young (1922) did not detect microfilariae in any of these animals and thus it is not possible for us to compare their prevalence data with the data we have collected for this study and thus to assess if prevalence levels of the parasite have changed in the last 100 years. The Gordon & Young (1922) survey nevertheless showed that *D. immitis* arrived in Manaus more than a century ago and, when taken together with our data, strongly suggest that the parasite has been endemic to Manaus ever since.

Consistent with what has been found in the Amazonas state municipality of Lábrea, we found that *D. immitis* prevalence levels were notably higher at our rural survey sites than they were at our urban sites (Soares et al., 2014). One possible explanation for this is that state-coordinated urban arbovirus control programs that have been in persistent action in Manaus for more than half a century (and aim to control dengue, zika and yellow fever transmission) have had a meaningful impact on the transmission on *D. immitis* in both Lábrea and Manaus. Studies in Africa have shown that in areas where *W. bancrofti* is transmitted by the malaria vector *Anopheles gambiae* and anti-malaria vector control methods are deployed, there has been significant reductions in *W. bancrofti* transmission even when *W. bancrofti* is not being specifically targeted for control (van den Berg et al., 2013). In Brazil the principal urban vector of *W. bancrofti* is *Culex quinquefasciatus* and is known to share breeding sites with the mosquitoes (*Aedes aegypti* and *Aedes albopictus*) that are targeted by Manaus´s arbovirus vector control programs (Consoli & Oliveira, 1994; Ahid & Lourençode-Oliveira, 1999; Padilla-Torres et al., 2013; Ríos-Velásquez et al., 2007). It may be, therefore, that these interventions have been responsible for a significant reduction of Manaus´s urban transmission of *W. bancrofti* and *D. immitis* which has resulted in the loss of *W. bancrofti* and kept *D. immitis* prevalence levels very low (Martins et al., 2021; Padilla-Torres et al., 2013; Ríos-Velásquez et al., 2007).

Beyond the veterinary health importance of our results for the companion animal owners of Manaus, our results are also of some importance for the Brazilian health service (SUS) and in particular for hospitals serving the rural areas of Manaus. While there have not been any formal reports of *D. immitis* occurring on CT and X-ray scans from the city, this does not mean that *D. immitis* larvae have not been detected in scans and public resources spent trying to determine if they are a symptom of a more serious lung disease. Our results suggest that clinicians working in endemic areas of Manaus should consider the possibility of *D. immitis* parasites when investigating coin-shaped lesions that appear on CT or X-ray scans.

There are many reliable and cheap PCR assays that can be used to identify *D. immitis* parasites from blood samples or biopsies, making these assays available to Manaus residents through public or private diagnostic service providers should thus now be encouraged (Simón et al., 2012, Dantas-Torres et al., 2017). A heightened awareness of the disease´s presence within Manaus should also be encouraged; there are effective treatments and prophylactics available for *D. immitis* infections and thus if Manaus veterinary practitioners and companion animal owners who live in the city are made aware of them the parasites transmission could be reduced (Simón et al., 2012, Turner et al., 2020). At present it is recommended that *D. immitis* infections are treated with a 28-day program of doxycycline in combination with ivermectin and melarsomine (Turner et al., 2020; Ta-Tang et al., 2021; Simón et al., 2012), however, it is very likely that faster-acting curative treatments for Dirofilariosis infections will soon be made available (Bakowski & McNamara, 2019; Turner et al., 2020). There is therefore also a need for Manaus´s veterinarians and companion animal owners to stay abreast of the latest developments in *D. immitis* treatment options. In Europe and North America there are heart worm societies where concerned companion animal owners liaise with professional veterinarians to monitor disease transmission and exchange up to date advice on diagnosis and treatment (Ta-Tang et al., 2021; Simón et al., 2012). At present there is no equivalent forum for native Portuguese speakers in Brazil; our study, however, makes clear the need for one.

Although non-human primate filarial parasites have been recently recorded in the state of Amazonas and indeed Manaus, as of yet, there have not been reports of these parasites, or indeed *D. immitis* parasites, causing disease in humans (Silva et al., 2022; Costa et al., 2023). In Pará state (Amazonas state´s eastern neighbor, which contains a similar Amazon-region rainforest ecology), however, there have been reports of zoonotic filarial parasites causing ocular infections (Bain et al., 2011; Otranto et al., 2011). Interestingly, molecular analysis found one of these Para state parasites to be similar but distinct from *D. immitis* parasites (Otranto et al., 2011). Future molecular studies on the *D. immitis* found in Manaus could thus help to determine if the *D. immitis* are like standard forms of *D. immitis* or more like those found in Para state and indeed what pathologies they are likely to cause in humans.

## References

[B001] Abrahim CMM, Py-Daniel V, Luz SLB, Fraiji NA, Stefani MMA (2019). Detection of *Mansonella ozzardi* among blood donors from highly endemic interior cities of Amazonas state, northern Brazil. Transfusion.

[B002] Ahid SMM, Lourenço-de-Oliveira R (1999). Mosquitoes potential vectors of canine heartworm in the Northeast Region from Brazil. Rev Saude Publica.

[B003] Bain O, Otranto D, Diniz DG, dos Santos JN, de Oliveira NP, Frota de Almeida IN (2011). Human intraocular filariasis caused by *Pelecitus* sp. Nematode, Brazil. Emerg Infect Dis.

[B004] Bakowski MA, McNamara CW (2019). Advances in antiwolbachial drug discovery for treatment of parasitic filarial worm infections. Trop Med Infect Dis.

[B005] Consoli RAGB, Oliveira RL (1994). Principais mosquitos de importância sanitária no Brasil..

[B006] Costa CHA, Crainey JLC, Vicente ACP, Conga DF, Gordo M, Luz SLB (2023). Ribosomal, mitochondrial and bacterial (*Wolbachia*) reference sequences for *Dipetalonema gracile* obtained from a wild pied tamarin (Saguinus bicolor) host in Manaus, Brazil. Acta Amazon.

[B007] Dantas-Torres F, Brianti E, Otranto D, Marcondes C. (2017). Arthropod borne diseases..

[B008] Dantas-Torres F, Otranto D (2020). Overview on *Dirofilaria immitis* in the Americas, with notes on other filarial worms infecting dogs. Vet Parasitol.

[B009] Gordon RM, Young CJ (1922). Parasites in dogs and cats in Amazonas. Ann Trop Med Parasitol.

[B010] Knight DH (1977). Heartworm heart disease. Adv Vet Sci Comp Med.

[B011] Martins M, Guimarães RCS, Fontes G (2021). Interruption of lymphatic filariasis transmission in Manaus, a former focus of *Wuchereria bancrofti* in the Western Brazilian Amazon. Rev Panam Salud Publica.

[B012] McCall JW, Genchi C, Kramer LH, Guerrero J, Venco L (2008). Heartworm disease in animals and humans. Adv Parasitol.

[B013] Otranto D, Diniz DG, Dantas-Torres F, Casiraghi M, de Almeida IN, de Almeida LN (2011). Human intraocular filariasis caused by *Dirofilaria* sp. Nematode, Brazil. Emerg Infect Dis.

[B014] Padilla-Torres SD, Ferraz G, Luz SLB, Zamora-Perea E, Abad-Franch F (2013). Modeling dengue vector dynamics under imperfect detection: three years of site-occupancy by *Aedes aegypti* and *Aedes albopictus* in urban Amazonia. PLoS One.

[B015] Peroba SC, Sperandio NC, Martins IVF (2022). Identificação e diferenciação morfológica de microfilárias no sangue de cães do Espírito Santo. Pubvet.

[B016] Ríos-Velásquez CM, Codeço CT, Honório NA, Sabroza PS, Moresco M, Cunha ICL (2007). Distribution of dengue vectors in neighborhoods with different urbanization types of Manaus, state of Amazonas, Brazil. Mem Inst Oswaldo Cruz.

[B017] Silva AMA, Almeida KS, Sousa JJN, Freitas FLC (2008). Canine dirofilariasis in Coari city, Amazonas State, Brazil. Arch Vet Sci.

[B018] Silva TRR, Narzetti LHA, Crainey JL, Costa CH, Santos YVS, Leles LFO (2022). Molecular detection of *Mansonella mariae* incriminates *Simulium oyapockense* as a potentially important bridge vector for Amazon-region zoonoses. Infect Genet Evol.

[B019] Simón F, Siles-Lucas M, Morchón R, González-Miguel J, Mellado I, Carretón E (2012). Human and animal dirofilariasis: the emergence of a zoonotic mosaic. Clin Microbiol Rev.

[B020] Soares HS, Camargo LMA, Gennari SM, Labruna MB (2014). Survey of canine tick-borne diseases in Lábrea, Brazilian Amazon: ‘accidental’ findings of *Dirofilaria immitis* infection. Rev Bras Parasitol Vet.

[B021] Ta-Tang TH, Luz SLB, Crainey JL, Rubio JM (2021). An overview of the management of mansonellosis. Res Rep Trop Med.

[B022] Turner JD, Marriott AE, Hong D, O’ Neill P, Ward SA, Taylor MJ (2020). Novel anti-*Wolbachia* drugs, a new approach in the treatment and prevention of veterinary filariasis?. Vet Parasitol.

[B023] van den Berg H, Kelly-Hope LA, Lindsay SW (2013). Malaria and lymphatic filariasis: the case for integrated vector management. Lancet Infect Dis.

